# Syndrome de sécrétion inappropriée d’hormone antidiurétique secondaire à la rispéridone et la carbamazépine: à propos d’un cas

**DOI:** 10.11604/pamj.2019.32.78.17720

**Published:** 2019-02-13

**Authors:** Assia Fadili, Nadia Attouche, Boubaker Charra, Khadija Mchichi Alami, Mohamed Agoub

**Affiliations:** 1 Centre Psychiatrique Universitaire Ibn Rochd, Casablanca, Maroc; 2 Service de Réanimation Médicale, CHU Casablanca, Maroc

**Keywords:** Syndrome de sécrétion inappropriée d´hormone antidiurétique, hyponatrémie, rispéridone, carbamazépine, Syndrome of inappropriate antidiuretic hormone secretion, hyponatraemia, risperidone, carbamazepine

## Abstract

Le syndrome de sécrétion inappropriée de l'hormone antidiurétique (SIADH) représenterait environ 50% de tous les cas diagnostiqués d'hyponatrémie. Seule une faible proportion de SIADH serait d'origine médicamenteuse. Nous rapportons le cas d'une patiente suivie pour un trouble schizo-affectif qui a développé le SIADH après avoir commencé un traitement à base de la rispéridone et la carbamazépine. Les résultats des tests biochimiques suggéraient un SIADH secondaire à l'utilisation de la rispéridone et la carbamazépine. La patiente a été traitée avec succès par l'arrêt des deux médicaments et une restriction hydrique. Après correction de la natrémie la décision thérapeutique était de mettre la patiente sous clozapine. Elle est actuellement sous 400mg avec des taux de natrémie stables. Les psychiatres doivent être conscients du risque d'hyponatrémie sévère associé aux médicaments psychotropes. Il est donc primordial de surveiller les électrolytes, en particulier le sodium, chez les patients prenant des antipsychotiques et des anticonvulsivants.

## Abstract (en)

The inappropriate antidiuretic hormone secretion Syndrome (ADHS) accounts for approximately 50% of all diagnosed cases of hyponatremia while drug-induced ADHS accounts for a small proportion of cases. We report the case of a female patient, treated for schizoaffective disorder, who developed ADHS following the initiation of risperidone and carbamazepine. Biochemical test results suggested risperidone and carbamazepine-induced ADHS. The patient was successfully treated by stopping drug use and by fluid restriction. After correcting the serum sodium levels, the patient was treated with clozapine. She is currently on clozapine 400mg with stable serum sodium rates. Psychiatrists should be aware of the risk of severe hyponatremia associated with psychotropic drug use. It is therefore essential to monitor electrolyte levels, in particular sodium levels, in patients taking antipsychotics and anticonvulsants.

## Introduction

Le syndrome de sécrétion inappropriée d'hormone antidiurétique (SIADH) se caractérise par la libération prolongée d'hormone antidiurétique (ADH) en l'absence de stimuli osmotique ou non osmotique ou par une action rénale renforcée de l'ADH. Il est caractérisé par une hyponatrémie de dilution (<135mmol/l), une augmentation du taux de sodium urinaire (> 20mmol/l), une osmolalité urinaire élevée (>100 mOsm/kg) par rapport à l'osmolalité plasmatique (< 280 mOsm/kg), chez des patients euvolémiques qui ne prenant aucun diurétique, avec des fonctions cardiaques, hépatiques, rénales, surrénaliennes et thyroïdiennes normales [1]. Le SIADH peut être induit par diverses affections, notamment des affections malignes, des maladies pulmonaires, des troubles du système nerveux central et de nombreux médicaments (vasopressine, desmopressine, ocytocine, antidépresseurs, antipsychotiques, carbamazépine) [1]. Le syndrome de sécrétion inappropriée de l'hormone antidiurétique (SIADH) représenterait environ 50% de tous les cas diagnostiqués d'hyponatrémie [2]. Seule une faible proportion de SIADH serait d'origine médicamenteuse [3]. Nous rapportons le cas d'une patiente qui a développé le SIADH après avoir commencé un traitement à base de la rispéridone et la carbamazépine.

## Patient et observation

La patiente âgée de 49 ans, mariée, sans enfants. Elle est connue malade mentale depuis 30 ans avec un suivi psychiatrique irrégulier et une mauvaise observance thérapeutique. Ses antécédents médicaux ne contenaient aucune autre maladie chronique, tumeur maligne ou traitement concomitant. La patiente a été admise au Service de Psychiatrie pour rechute de son trouble schizo-affectif, avec une hétéro-agressivité physique et trouble de l'ordre public. Elle a été mise sous rispéridone et carbamazépine, avec une augmentation progressive des doses. Un bilan biologique a été demandé à l'admission fait d'ionogramme sanguin, la numération de la formule sanguine, bilan hépatique et rénale, s'est révélé normal. A une semaine de traitement, lors des analyses biologiques de contrôle, on découvre une hyponatrémie à 128mmol/L sans signe d'appel clinique, le reste du bilan biologique était normal. La patiente était mise sous 8mg de rispéridone et 1200mg de carbamazépine, la décision thérapeutique était d'arrêter la carbamazépine et de réduire la dose de la rispéridone avec un contrôle biologique ultérieur. A 10 jours du traitement, les analyses biologiques ont objectivé: une natrémie sérique à 119mmol/L, le taux de potassium sérique à 4,56mmol/L, une osmolalité plasmatique à 246 mOsm/Kg, une osmolalité urinaire à 260mOsm/Kg, le taux de sodium urinaire est de 40mEq/L.

La numération de la formule sanguine, la glycémie, l'albumine, les tests de la fonction hépatique et rénale ainsi que la protéine C réactive (CRP) étaient normaux. Cliniquement, la patiente se plaignait d'une fatigue et de somnolence. Elle était apyrétique et stable sur le plan hémodynamique, respiratoire et neurologique. Il n'y avait pas de signes cliniques d'hypo ou d'hyper-volémie, notamment pas d'œdème périphérique et absence de signes de déshydratation. De même, il n'y avait aucune preuve d'absorption excessive de liquide ou de polydipsie psychogène. Dans le but de clarifier le diagnostic, un ensemble d'investigations biologiques et radiologiques a été demandé: les tests de la fonction thyroïdienne ainsi que le taux de cortisol à 8 heures étaient dans les limites normales. Les sérologies hépatiques, syphilitiques, et du VIH sont revenues négatives. En outre, l'électrocardiogramme, la radiographie thoracique, et la tomodensitométrie cérébrale n'ont révélé aucune anomalie. Compte tenu du tableau clinique et des résultats biologiques, le diagnostic du syndrome de sécrétion inappropriée d'hormone antidiurétique (SIADH) a été évoqué. L'origine médicamenteuse a été suspectée, ceci en absence de signes cliniques ou biologiques en faveur d'une autre cause étiologique néoplasique, infectieuse, inflammatoire, ou neurologique.

La conduite à tenir fut l'arrêt de la rispéridone et de la carbamazépine. Devant l'hyponatrémie sévère, un transfert dans un Service de Réanimation a été effectué. La patiente a séjourné pendant 48 heures, des mesures thérapeutiques ont été prises y compris la restriction liquidienne et l'ajout de soluté hypertonique (NaCl 3%) avec une surveillance biologique. Apres correction de la natrémie sérique à 134 mmol/L, la patiente nous a été adressée pour complément de sa prise en charge psychiatrique. Nous avons opté d'éviter de réintroduire la rispéridone et la carbamazépine et de démarrer la clozapine en augmentant progressivement les doses. Les taux de la natrémie sont restés stables, les analyses biologiques se situaient toutes dans la fourchette normale. Depuis sa sortie de l'hôpital, la patiente a un suivi psychiatrique régulier, avec stabilisation de son état psychiatrique et physique. Elle est actuellement à 8 mois de traitement sous 400mg de clozapine avec un bilan biologique normal.

## Discussion

Le taux de SIADH induit par les médicaments est relativement faible. Ce type de SIADH est le plus souvent réversible à l'arrêt de l'agent causal. Il est donc primordial d'évaluer le lien entre le SIADH et le médicament soupçonné [4]. L'évaluation optimale du lien entre un médicament et le SIADH passe par un diagnostic clair de SIADH à l'aide des critères diagnostiques essentiels. Il est également important d'éliminer toutes les autres causes possibles [3]. Dans notre cas l'installation du syndrome de sécrétion inappropriée d'hormone antidiurétique a fait suite à l'introduction de la rispéridone et la carbamazépine, en absence de signes cliniques ou biologiques en faveur d'une autre cause étiologique. En outre, la correction de la natrémie était suite à l'arrêt des deux psychotropes ce qui est en faveur de l'origine médicamenteuse. Les antipsychotiques, à la fois typiques et atypiques, pourraient causer le SIADH [5]. Le mécanisme par lequel les antipsychotiques provoquent le SIADH n'est pas encore clair. Diverses hypothèses ont été suggérées, notamment un blocage à long terme des récepteurs D2 conduisant à une hypersensibilité aux récepteurs D2, qui entraîne à son tour la libération de l'ADH [6, 7].

La carbamazépine est parmi les médicaments rapportés pour être liés à un nombre plus élevé de cas de SIADH [3]. La base physiopathologique exacte du SIADH induit par un médicament n'est toujours pas claire. La stimulation de la libération d'ADH et l'augmentation de son action sur les reins seraient les mécanismes les plus probables d'hyponatrémie induite par le médicament [1]. La prise en charge de l'hyponatrémie d'origine médicamenteuse secondaire à SIADH nécessite l'arrêt immédiat de l'agent en cause. En cas d'hyponatrémie sévère, des actions supplémentaires telles que la restriction liquidienne et l'administration de furosémide doivent être prises [1]. La mesure thérapeutique prise, dans le cas de notre patiente, est l'arrêt de toute médication avec une restriction liquidienne. La clozapine est probablement le seul antipsychotique à avoir un effet bénéfique sur le comportement polydipsique et le développement de l'hyponatrémie peut être dû à sa plus faible affinité de liaison aux récepteurs D2 [6-8]. En effet, après correction de la natrémie notre décision thérapeutique était de mettre la patiente sous clozapine. Elle est actuellement à 8 mois de traitement sous 400mg avec des taux de natrémie stables.

## Conclusion

Le cas que nous rapportons met en évidence le risque de développement du SIADH suite à l'utilisation de la rispéridone et la carbamazépine chez une patiente atteinte de psychose. Les psychiatres doivent être conscients du risque d'hyponatrémie sévère associé aux médicaments psychotropes. Il est donc primordial de surveiller étroitement les électrolytes, en particulier le sodium, chez les patients prenant des antipsychotiques et des anticonvulsivants. Les patients et les membres de leur famille doivent être informés des symptômes de l'hyponatrémie et il convient également de souligner l'importance de l'identification précoce de l'hyponatrémie [9].

## Conflits d’intérêts

Les auteurs ne déclarent aucun conflit d'intérêts.
